# Management of Life-Threatening Bleeding in Patients With Mechanical Heart Valves

**DOI:** 10.7759/cureus.15619

**Published:** 2021-06-13

**Authors:** Syed A Huda, Sara Kahlown, Mohammad H Jilani, Debanik Chaudhuri

**Affiliations:** 1 Internal Medicine, State University of New York (SUNY) Upstate Medical University, Syracuse, USA; 2 Internal Medicine, United Health Services Wilson Medical Center, Johnson City, USA; 3 Medicine, Carle Foundation Hospital, Urbana, USA; 4 Interventional Cardiology, State University of New York (SUNY) Upstate Medical University, Syracuse, USA

**Keywords:** mechanical heart valve, hemorrhage, intracranial bleeding, warfarin, valve thrombosis

## Abstract

Valvular heart disease is common in the United States, with a number of patients undergoing valve replacement procedures every year. The two types of valve prostheses include mechanical and bioprosthetic valves. Mechanical heart valves require lifelong anticoagulation with vitamin K antagonists like warfarin. The clinicians are often faced with the dilemma of major bleeding episodes such as intracranial hemorrhage or gastrointestinal bleeding in these patients. The management includes reversing warfarin-induced coagulopathy with vitamin K supplementation, fresh frozen plasma, or prothrombin complex concentrate (PCC), with PCC being the treatment of choice. With regard to the safe resumption of anticoagulation, guidelines are silent, and data is limited to case reports/series. This article reviews the present literature for the management of bleeding in patients with mechanical heart valves and the safe duration for holding off anticoagulation with minimal risk of valve thrombosis/thromboembolism.

## Introduction and background

Epidemiology of valvular heart disease

Valvular heart disease (VHD) is common in the United States (US) and its incidence increases with age with an estimated prevalence of 2.5%. Aortic stenosis, functional mitral regurgitation, and tricuspid regurgitation are the most common VHDs in the elderly population [1]. VHD usually has a prolonged asymptomatic period before patients develop symptoms. Definitive treatment includes transcatheter or surgical valve repair or replacement [2]. Around 100,000 patients undergo valve replacement procedures in the US every year [3].

The two types of commercially available valve prostheses include mechanical and bioprosthetic valves. The choice of valve depends on the underlying indication, suitability for anticoagulation, valve longevity, and patient’s preference [4]. Bioprosthetic valves are usually made from bovine pericardium or porcine aortic valves that undergo normal degenerative changes. Therefore, they last for only 10-15 years, often requiring reoperation. However, the main advantage of bioprosthetic valves is that they do not require long-term anticoagulation. On the other hand, the structural degeneration of mechanical valves is rare but they are highly thrombogenic requiring lifelong anticoagulation with vitamin K antagonists (VKAs) such as warfarin [5].

Risk of Thromboembolism

The risk of thromboembolism after valve replacement depends on the type of mechanical prosthesis used and the anatomical position. The three basic types of mechanical valves include caged ball, tilting disk, and bileaflet valve. The cage ball valve is the most thrombogenic while bileaflet valves have a low risk of thrombosis. Similarly, valves implanted in the mitral area confer a greater risk of thromboembolism given slower blood flow across the mitral orifice [6]. In the absence of antithrombotic therapy in patients with mechanical heart valves, the risk of major embolism (defined as peripheral ischemia requiring surgery, residual neurological deficit, or death) is about 4 per 100 patient-years [7]. This risk reduces to 2.2 per 100 patient-years with only oral antiplatelets and is further decreased to 1 per 100 patient-years with oral anticoagulants. Additional risk factors for thromboembolic events include atrial fibrillation, previous thromboembolism, left ventricular dysfunction, or hypercoagulable state [8].

## Review

Anticoagulation and its challenges

Warfarin

Warfarin is an oral anticoagulant that blocks the vitamin K-dependent gamma-carboxylation of coagulation factors II, VII, IX, and X and anticoagulant factors proteins C and S. The response to warfarin is monitored with the international normalized ratio (INR). It takes 24-36 hours after the first dose to notice a change in the INR [9]. There are no dosage recommendations for initiating therapy and treatment should be tailored for each patient with serial monitoring of the INR. It is preferable to specify a single INR target because it reduces the likelihood of INR values consistently near the upper or lower boundary of the range. Patients with mechanical aortic valves in the absence of risk factors of thromboembolism should have a target INR of 2.5 (2.0-3.0), whereas patients with mechanical aortic valves with additional risk factors for thromboembolism (atrial fibrillation, previous thromboembolism, left ventricular [LV] systolic dysfunction, or hypercoagulable condition) and mechanical aortic valves should have a target INR of 3 (2.5-3.5) [10]. Warfarin has a very narrow therapeutic index and the INR is affected by different factors including genetic polymorphism in warfarin metabolism, interaction with food and drugs, and patient compliance. Pokorney et al. found that only 59% of patients with atrial fibrillation in the US Outcomes Registry for Better Informed Treatment of Atrial Fibrillation (ORBIT-AF) had INR in the therapeutic range [11]. All these factors make the management of warfarin therapy a challenge for both the clinician and patient.

Risk of Bleeding

The Control of Anticoagulation Subcommittee of the International Society on Thrombosis and Hemostasis defines major bleeding in a non-surgical patient as (i) fatal bleeding and/or (ii) symptomatic bleeding in a critical area or organ, such as intracranial, intraspinal, intraocular, retroperitoneal, intra‐articular or pericardial, or intramuscular with compartment syndrome, and/or (iii) bleeding causing a fall in the hemoglobin level by 2 g/l or more, or leading to transfusion of two or more units of whole blood or red cells [12].

The incidence of major bleeding in patients with mechanical heart valves ranges from 0.34 to 2.91 per 100 patient-years [13-15]. The traditional risk factors include supratherapeutic INR, age >75 years, hypertension, previous stroke, concomitant antiplatelet use, and a prior history of bleeding [16]. The common sites of bleeding include the gastrointestinal tract, urinary tract, intracranial hemorrhage/subdural hematoma, and retroperitoneal hemorrhage.

Treatment of warfarin-induced coagulopathy

Vitamin K

Vitamin K is a specific reversal agent that restores the activity of the hepatic enzyme, vitamin K epoxide reductase in a dose-dependent manner. It can be given orally, subcutaneously, or intravenously. Slow intravenous administration (in 25-50 ml normal saline over 15-30 minutes) is preferred over oral or subcutaneous route given a more predictable response and rapid reduction of INR within four to six hours. Anaphylactic reactions are usually rare with current formulations [17].

According to 2020 American College of Cardiology (ACC)/American Heart Association (AHA) Guidelines, patients with mechanical heart valves and supratherapeutic INR (>5.0) who are not actively bleeding are not likely to benefit from routine vitamin K administration in addition to temporary VKA cessation. This recommendation is based on the systematic review that indicated a nonsignificant increased risk of mortality and thromboembolism with vitamin K administration, with only moderate certainty of the evidence [18]. For patients with life-threatening bleeding, who are receiving the 4-factor prothrombin complex concentrate (4F-PCC), a 10-mg intravenous dose of vitamin K is only recommended when there is no plan for restarting anticoagulation within the next week [19].

Fresh Frozen Plasma

Fresh frozen plasma (FFP) is derived from the human blood and contains all the coagulation factors and proteins. It requires the ABO blood group type match and it must be thawed before administration that can lead to a time lag of up to 90 minutes between placing the order and the patient receiving it; 1 unit of FFP contains 1 unit of the given coagulation factors, so a plasma dose of 15-30 ml/kg is required for adequate VKA reversal, but it is not practical given such a large volume. Usually, a plasma concentration of 10-15 ml/kg is used in clinical practice. Potential adverse effects of plasma transfusion include risk of pathogen transmission, allergic reactions, fluid overload, and risk of transfusion-related lung injury [20].

Prothrombin Complex Concentrate

PCC is extracted from the human plasma and it only contains purified vitamin K-dependent clotting factors. Nonactivated 3-factor (3F) PCCs have FII, FIX, and FX with negligible FVII and proteins C and S, while nonactivated 4F-PCCs contain FII, FVII, FIX, FX, and proteins C and S. They are stored as a lyophilized powder at room temperature and do not require ABO blood group compatibility. Only 4F-PCC is Food and Drug Administration (FDA) approved for warfarin reversal and it contains 25 times the concentration of vitamin K-dependent factors per unit volume as compared to plasma. Therefore, 4F-PPC is preferred over plasma given smaller volumes and much faster infusion rate. The dosage is based on INR and body weight. The recommended doses are as follows: for INR 2 to <4, 25 U/kg; for INR 4-6, 35 U/kg; for INR >6, 50 U/kg; maximum dose is 5000 units capped at 100 kg body weight. An alternate regimen involves a fixed dose of 1000 units for any non-intracranial bleeding and 1500 units for intracranial hemorrhage [21].

There is a concern for an increased risk of thromboembolism with PCC use based on anecdotal data suggesting an increased risk of thromboembolic events in patients with hemophilia who received frequent 3F/4F-PCCs. However, recent trials comparing 4F-PCC with plasma for VKA reversal showed comparable thromboembolic events in both groups [22].

How Long Can Warfarin Be Safely Held Off?

The clinicians are often faced with the dilemma of managing anticoagulation in patients with mechanical heart valves who present with major hemorrhage. There are no large prospective trials to guide in this regard. We reviewed previous studies involving patients with mechanical heart valves who had major bleeding episodes and the results are summarized in Table 1.

**Table 1:** Summary of case series reporting IC hemorrhage in patients with mechanical heart valves IC, intracranial; GI, gastrointestinal; VTE, venous thromboembolism

Authors	Number of Participants	Valve Position					Type of Hemorrhage					Duration Off Anticoagulation (Days)	VTE	Recurrent Hemorrhage
		Mitral		Aortic		Both	IC	GI			Other			
Wijdicks et al. [23]	39	20	16		30		39		0	0		2-90 (median 8)	0	0
Ananthasubramanian et al. [24]	28	12	12		4		3		28	0		15 ± 4	0	10
Krittalak et al. [25]	26	15	6		5		26		0	0		8.5 ± 7.7	2	Not reported
Amin et al. [26]	12	1	11		0		12		0	0		9 ± 8.7	0	2
Kuramatsu et al. [27]	137	47	90		0		137		0	0		Not reported	8	21

In an observational study involving 137 patients with mechanical heart valves who presented with intracranial hemorrhage, Kuramatsu et al. observed that holding off anticoagulation for two weeks is safe and its earlier resumption should only be considered in high-risk patients [27]. Similarly, Amin et al. retrospectively observed 12 patients with mechanical heart valves who had surgical treatments for subdural hematoma and were followed up for a mean duration of 50 months [26]. In this study, anticoagulation was held off for an average of two weeks. No death or thromboembolic event occurred during this time and only two patients had an episode of recurrent subdural hematoma.

Phan et al. retrospectively evaluated the management of anticoagulation in 52 patients with mechanical heart valves and intracranial bleeding and found that warfarin was restarted in 28 patients by day 30 without the recurrence of intracranial hemorrhage [28]. The authors found that the cumulative risk of ischemic stroke at 30 days in patients with a metallic valve was 3% (95% CI, 0%-8%). However, 40% of the patients in this study group had died by day 30, so it is difficult to conclude the safe duration for holding off anticoagulation. In another study, Wijdicks et al. concluded that in patients with mechanical heart valves without any prior history of systemic embolization, anticoagulation could be safely withheld for one to two weeks with minimal risk of thromboembolism [23].

In a systematic review, Romualdi et al. evaluated the optimal timing to restart anticoagulation after intracranial hemorrhage and concluded that anticoagulation can be safely withheld for 14 days [29]. Conversely in a meta-analysis, AlKherayf et al. opined that there is a lack of quality data to suggest the optimal timing to restart anticoagulation following an intracranial hemorrhage [30].

To Bridge or Not to Bridge

There is a dearth of robust data to suggest a considerable decrease in the incidence of thromboembolism with bridging therapy, and most recent studies have pointed towards the increased frequency of major bleeding as compared to thromboembolism [31]. Schulman et al. retrospectively observed 117 patients who underwent bridging with low-molecular-weight heparin (LMWH) for invasive procedures. Bleeding was more common in the bridging arm, but there was no episode of thromboembolism in either group. Interestingly, they found that surgery alone was an independent risk factor for bleeding and not the timing or dosage of the LMWH [32]. In a similar prospective study involving 556 participants with mechanical heart valves undergoing elective procedures, Daniels et al. looked at the incidence of thromboembolism after anticoagulation was temporarily held off. The authors found a higher three-month cumulative incidence of major bleeding (3.6%) as compared to thromboembolism (0.9%) [33]. ACC/AHA guidelines also recommend periprocedural bridging therapy in patients with mechanical mitral valves or mechanical aortic valves and for any thromboembolic risk factor on an individualized basis after weighing the risk of bleeding against the benefit of thromboembolism prevention [19]. Although these studies did not include patients with bleeding, clinicians should seriously consider bridging patients with a high risk of thromboembolism with unfractionated heparin or LMWH after weighing risk versus benefits.

## Conclusions

In conclusion, all guidelines recommend early resumption of anticoagulation but do not provide guidance about the safe duration to hold it off. There are no clinical trials or prospective studies to guide in such situations either and the literature is limited to case reports and series. The available data suggests that in patients who present with major hemorrhage, INR should be reversed with PCC, and vitamin K be only administered if there is no plan to restart anticoagulation within the next week. Warfarin can safely be held off for one to two weeks in these patients with minimal risk of thromboembolism, whereas in patients with mechanical mitral valves or mechanical aortic valves with risk factors for thromboembolism, therapeutic anticoagulation should be started as soon as felt safe by the clinician, and they might also benefit from bridging therapy while the INR is subtherapeutic.

## References

[1] Nkomo VT, Gardin JM, Skelton TN, Gottdiener JS, Scott CG, Enriquez-Sarano M (2006). Burden of valvular heart diseases: a population-based study. Lancet.

[2] Moore M, Chen J, Mallow PJ, Rizzo JA (2016). The direct health-care burden of valvular heart disease: evidence from US national survey data. Clinicoecon Outcomes Res.

[3] Lloyd-Jones D, Adams RJ, Brown TM (2010). Executive summary: heart disease and stroke statistics—2010 update: a report from the American Heart Association. Circulation.

[4] Catterall F, Ames PR, Isles C (2020). Warfarin in patients with mechanical heart valves. BMJ.

[5] Head SJ, Çelik M, Kappetein AP (2017). Mechanical versus bioprosthetic aortic valve replacement. Eur Heart J.

[6] Leiria TL, Lopes RD, Williams JB, Katz JN, Kalil RA, Alexander JH (2011). Antithrombotic therapies in patients with prosthetic heart valves: guidelines translated for the clinician. J Thromb Thrombolysis.

[7] Cannegieter SC, Rosendaal FR, Briët E (1994). Thromboembolic and bleeding complications in patients with mechanical heart valve prostheses. Circulation.

[8] Poli D, Antonucci E, Pengo V (2018). Mechanical prosthetic heart valves: quality of anticoagulation and thromboembolic risk. The observational multicenter PLECTRUM study. Int J Cardiol.

[9] Kuruvilla M, Gurk-Turner C (2001). A review of warfarin dosing and monitoring. Proc (Bayl Univ Med Cent).

[10] Otto CM, Nishimura RA, Bonow RO (2021). 2020 ACC/AHA guideline for the management of patients with valvular heart disease: executive summary: a report of the American College of Cardiology/American Heart Association Joint Committee on Clinical Practice Guidelines. Circulation.

[11] Pokorney SD, Simon DN, Thomas L (2015). Patients' time in therapeutic range on warfarin among US patients with atrial fibrillation: results from ORBIT-AF registry. Am Heart J.

[12] Schulman S, Kearon C, Subcommittee on Control of Anticoagulation of the Scientific and Standardization Committee of the International Society on Thrombosis and Haemostasis (2005). Definition of major bleeding in clinical investigations of antihemostatic medicinal products in non-surgical patients. J Thromb Haemost.

[13] Akhtar RP, Abid AR, Zafar H, Khan JS (2009). Aniticoagulation in patients following prosthetic heart valve replacement. Ann Thorac Cardiovasc Surg.

[14] Labaf A, Svensson PJ, Renlund H, Jeppsson A, Själander A (2016). Incidence and risk factors for thromboembolism and major bleeding in patients with mechanical valve prosthesis: a nationwide population-based study. Am Heart J.

[15] Hering D, Piper C, Bergemann R, Hillenbach C, Dahm M, Huth C, Horstkotte D (2005). Thromboembolic and bleeding complications following St. Jude Medical valve replacement: results of the German Experience With Low-Intensity Anticoagulation Study. Chest.

[16] Schulman S, Beyth RJ, Kearon C, Levine MN (2008). Hemorrhagic complications of anticoagulant and thrombolytic treatment: American College of Chest Physicians Evidence-Based Clinical Practice Guidelines (8th Edition). Chest.

[17] Britt RB, Brown JN (2018). Characterizing the severe reactions of parenteral vitamin K1. Clin Appl Thromb Hemost.

[18] Khatib R, Ludwikowska M, Witt DM (2019). Vitamin K for reversal of excessive vitamin K antagonist anticoagulation: a systematic review and meta-analysis. Blood Adv.

[19] Otto CM, Nishimura RA, Bonow RO (2021). 2020 ACC/AHA guideline for the management of patients with valvular heart disease: a report of the American College of Cardiology/American Heart Association Joint Committee on Clinical Practice Guidelines. Circulation.

[20] Sarode R, Milling TJ Jr, Refaai MA, Mangione A, Schneider A, Durn BL, Goldstein JN (2013). Efficacy and safety of a 4-factor prothrombin complex concentrate in patients on vitamin K antagonists presenting with major bleeding: a randomized, plasma-controlled, phase IIIb study. Circulation.

[21] Tomaselli GF, Mahaffey KW, Cuker A (2020). 2020 ACC expert consensus decision pathway on management of bleeding in patients on oral anticoagulants: a report of the American College of Cardiology Solution Set Oversight Committee. J Am Coll Cardiol.

[22] Milling TJ Jr, Refaai MA, Sarode R (2016). Safety of a four-factor prothrombin complex concentrate versus plasma for vitamin K antagonist reversal: an integrated analysis of two phase IIIb clinical trials. Acad Emerg Med.

[23] Wijdicks EF, Schievink WI, Brown RD, Mullany CJ (1998). The dilemma of discontinuation of anticoagulation therapy for patients with intracranial hemorrhage and mechanical heart valves. Neurosurgery.

[24] Ananthasubramaniam K, Beattie JN, Rosman HS, Jayam V, Borzak S (2001). How safely and for how long can warfarin therapy be withheld in prosthetic heart valve patients hospitalized with a major hemorrhage?. Chest.

[25] Krittalak K, Sawanyawisuth K, Tiamkao S (2011). Safety of withholding anticoagulation in patients with mechanical prosthetic valves and intracranial haemorrhage. Intern Med J.

[26] Amin AG, Ng J, Hsu W, Pradilla G, Raza S, Quinones-Hinojosa A, Lim M (2013). Postoperative anticoagulation in patients with mechanical heart valves following surgical treatment of subdural hematomas. Neurocrit Care.

[27] Kuramatsu JB, Sembill JA, Gerner ST (2018). Management of therapeutic anticoagulation in patients with intracerebral haemorrhage and mechanical heart valves. Eur Heart J.

[28] Phan TG, Koh M, Wijdicks EF (2000). Safety of discontinuation of anticoagulation in patients with intracranial hemorrhage at high thromboembolic risk. Arch Neurol.

[29] Romualdi E, Micieli E, Ageno W, Squizzato A (2009). Oral anticoagulant therapy in patients with mechanical heart valve and intracranial haemorrhage. A systematic review. Thromb Haemost.

[30] AlKherayf F, Xu Y, Gandara E, Westwick H, Moldovan ID, Wells PS (2016). Timing of vitamin K antagonist re-initiation following intracranial hemorrhage in mechanical heart valves: systematic review and meta-analysis. Thromb Res.

[31] Tan CW, Wall M, Rosengart TK, Ghanta RK (2019). How to bridge? Management of anticoagulation in patients with mechanical heart valves undergoing noncardiac surgical procedures. J Thorac Cardiovasc Surg.

[32] Schulman JM, Majeed A, Mattsson E, Schulman S, Holmström M, Ågren A (2015). Strategies and outcomes of periprocedural bridging therapy with low-molecular-weight heparin in patients with mechanical heart valves. J Thromb Thrombolysis.

[33] Daniels PR, McBane RD, Litin SC, Ward SA, Hodge DO, Dowling NF, Heit JA (2009). Peri-procedural anticoagulation management of mechanical prosthetic heart valve patients. Thromb Res.

